# Post and Core for Telescopic Crown-Retained Dentures—An In Vitro Comparison of Different Materials Using Chewing Simulation

**DOI:** 10.3390/dj14040233

**Published:** 2026-04-14

**Authors:** Jonas Adrian Helmut Vogler, Milan Rachold, Bernd Wöstmann, Peter Rehmann, Kay-Arne Walther

**Affiliations:** Dental Clinic—Department of Prosthodontics, Justus Liebig University, Schlangenzahl 14, 35392 Giessen, Germany

**Keywords:** post and core, telescopic denture, CAD/CAM, chewing simulation

## Abstract

**Objectives:** Due to extra-axial forces, post and core (PC) treatment has the worst survival probability in abutment teeth for telescopic crown-retained dentures (TCDs). The reason for this is a mismatch regarding the mechanical properties between PC material and dentin or a poor accuracy of fit of PC, resulting in tooth fracture or decementation. However, the inclusion of severely damaged endodontically treated teeth needing PC is often mandatory in order to achieve a stable situation for TCD. Thus, an advancement of PC treatment for TCD is of high clinical interest. Recently it has become possible to fabricate customized PC with favourable mechanical properties by using CAD/CAM technology. **Methods:** Thus, the aim of this investigation was to compare the performance of these PC types (CAD/CAM PC) to customized cast PC (CPC) and prefabricated fibre-reinforced PC (PFPC) in a TCD set-up using a chewing simulator. **Results:** The investigation group with CAD/CAM PC showed neither tooth fracture nor decementation, in contrast to the CPC and PFPC groups, in which both types of failure were recorded. Thus, CAD/CAM PC showed significantly better performance than CPC and PFPC. **Conclusions:** Within the limitations, CAD/CAM PCs are therefore recommendable for PC treatment with TCD.

## 1. Introduction

Telescopic crown-retained dentures (TCD) are a common treatment option in cases with only few remaining teeth [1,2]. However, in many cases it is mandatory to include severely damaged endodontically treated teeth needing PC in order to obtain a stable support for the denture [3]. However, in scientific dental literature, many authors describe significant differences between survival of abutment teeth with and without PC that should be considered when it comes to treatment planning for TCD [4]. Furthermore, the majority of studies investigating the survival probability of PC considering different covering prosthetic restorations found the worst results for abutment teeth of TCDs [4,5]. This applies both to the survival probability of PC itself [6] and to the risk of extraction of the abutment tooth [7]. The main reasons for this are lever forces that occur when TCD is inserted and removed wrongly by the patient or when the saddle is incongruent to the tissue [5]. Due to the rigid connection between the abutment tooth and the denture [3], the occurrence of these forces is also responsible for the increased risk of root fracture, especially in the first five years after fitting of PC [7], and is even further exacerbated if the mechanical properties of the PC material does not match those of dentin [8,9,10]. In particular, this is the case with customized cast PC (CPC) that has been the gold standard for PC treatment during recent decades [8]. That is why many authors now prefer the use of prefabricated fibre-reinforced PCs (PFPCs) as they have a dentin-like elastic modulus and beyond that can be fitted in a single session [10,11,12]. However, it has to be kept in mind that PFPCs have a poorer fit in the prepared root canal compared to customized PCs [13]. This is particularly relevant in the context of the extra-axial forces linked to TCD as it can lead to an increased risk of decementation [14]. Moreover, poor accuracy of fit of PC is associated with an increased risk of secondary caries, and thus an increased risk of tooth loss [14]. Furthermore, due to polymerization shrinkage, a thick resin layer between the post and the root canal wall can lead to uneven force transmission into the root dentin with an associated increased risk of root fracture [13,15,16].

In the context of the occurrence of extra-axial forces with TCD, fracture of abutment teeth without PC or even without endodontical treatment is commonly described as the most frequent cause of failure with TCDs [3,17]. However, this type of failure is not mandatorily connected to the extraction of the tooth [18], because the fracture line often crosses the area of tooth preparation, leaving out the root but resulting in insufficient surface to refit the telescopic crown without PC fitting [19]. In these cases, PC can be used to refit the telescopic crown on the abutment tooth in order to recover the function of TCDs [20]. However, the long-term survival of this treatment using CPC and PFPC is not satisfactory [21]. Therefore, further development of PC treatment especially for abutment teeth of TCDs is of high clinical interest [21].

The latest technical advances in intraoral scanners and digital manufacturing have made it possible to scan the post space preparation and fabricate customized PC in a totally digital CAD/CAM workflow [13]. Firstly, this workflow increases the accuracy of fit not only compared to PFPC but also compared to analogue-manufactured CPC [22]. Secondly, the mechanical properties of the manufactured PC can be improved because there are dentin-like materials that are limited to CAD/CAM fabrication and show favourable results in anterior teeth with extra-axial forces, resulting in decreased risk of root fracture and decementation [8]. Summarizing these results, the advantages of CPC and PFPC can be combined in CAD/CAM PC by using modern digital technologies; thus, PC treatment of TCD could be improved by this workflow as well. Thus, the aim of the present investigation was to compare the performance of CPC, PFPC and CAD/CAM PC in a TCD set-up using artificial alteration in a chewing simulator. The following null hypotheses were put forward in order to address the research issue:

– There are no significant differences between CPC, PFPC and CAD/CAM PC regarding root fracture.
– There are no significant differences between CPC, PFPC and CAD/CAM PC regarding decementation of PC.

## 2. Materials and Methods

For the statistical calculation of the significant sample size, a power analysis [23] with a pursued power of 95% was conducted using data from a comparable study [8]. In this previous study, 23% of CPCs showed failure whereas no CAD/CAM PCs lost retention or led to root fracture during chewing simulation. Therefore, the assumed difference in failure rates between the investigation groups of this study was 23%, resulting a calculated sample size of 21 per investigation group. Nevertheless, we considered that the data in the scientific dental literature is scarce on this rather novel topic. Thus, we increased the sample size to 30 teeth per investigation group (CPC, PFPC and CAD/CAM PC) in order to ensure statistical significance. Table 1 shows the materials used in this study along with the elastic modulus for CPC, PFPC and CAD/CAM PC fabrication in relation to the elastic modulus of dentin.

For this study, 90 comparable human lower canines were used. The teeth were stored in physiological saline solution in a refrigerator at 8 °C, which is a common procedure in the scientific dental literature [25,26,27,28]. A root size of 15 mm length and 6 mm width (±1.0 mm) [29,30] was determined and all teeth were extracted for therapeutic reasons. The ethics committee of the Medical Faculty of Justus-Liebig University Giessen, Germany *[removed for anonymity reasons]* approved the use of the teeth for research purposes. Before using the teeth for the present investigation, the roots were visually checked for the absence of fractures or cracks under a digital light microscope (Smartzoom 5, Zeiss, Jena, Germany) and by X-ray. With a disk grinder under water cooling, the crowns were removed following an endodontic treatment using F-360 (ISO 15-45, Komet, Lemgo Germany) and 3.0% sodium hypochlorite. Subsequently, the prepared root canal was filled with gutta-percha (ISO 45, Taper 0.04, Coltène/Whaledent AG, Altstätten Switzerland) and sealer (AH Plus, Dentsply DeTrey, Bensheim, Germany), using the one-point technique. The following post space preparation was conducted after the sealer was totally cured (24 h) by use of the ER System (ISO 90, Komet, Lemgo, Germany) at a length of 10 mm.

**Figure 1:**
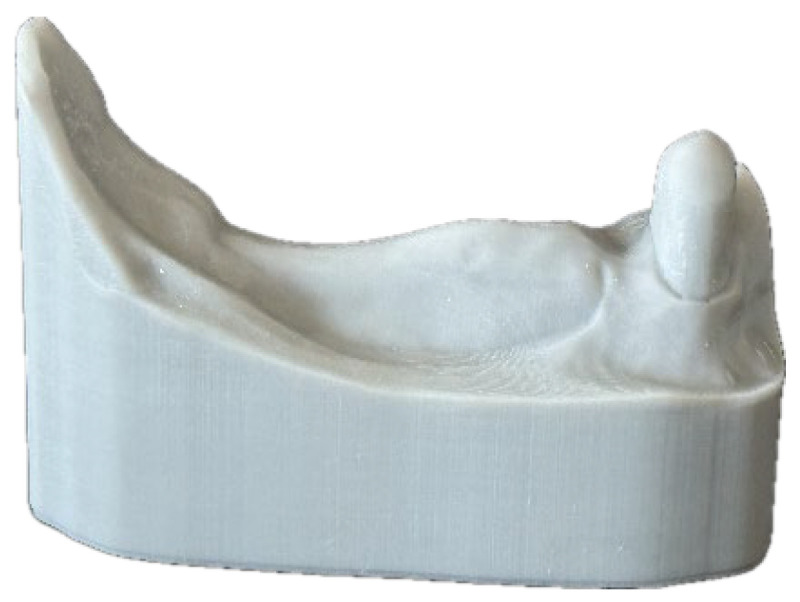
3D-printed model of the tissue for TCD with abutment tooth 43.

Furthermore, 90 identical lower jaw models of the tegument for TCD with abutment tooth 43 were 3D-printed (SHERAPrint 30, Shera, Lemförde, Germany) out of a resin for dental models (SHERAmodel stone, Shera, Lemförde, Germany) (Figure 1).

Subsequently, the abutment tooth was removed from the model and a hole was drilled to fit the root of the human lower canine using impression material (Impregum, 3M, Neuss, Germany) as a simulated periodontal ligament. Using Periotest (Periotest Classic type 3218, Medizintechnik Gulden e.K.,, Modautal, Germany) three measurements were processed in order to ensure that the specimen was equivalent to an abutment tooth with mobility grade 0. When the mean value was outside the range of −08 to +09, the specimen was excluded from further investigation and the amount of sample teeth was increased in order to maintain the initial sample size of 90 teeth for this study.

**Figure 2:**
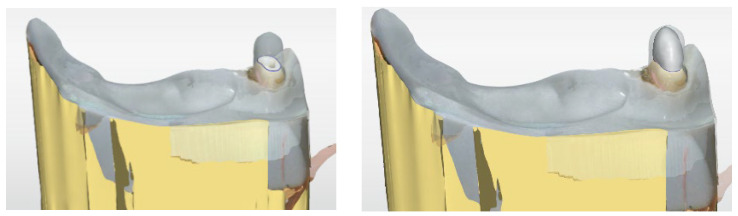
(**Left**): Digital impression of the model with canine and superimposition of the dataset for model printing. (**Right**): Digital construction for CPC and CAD/CAM PC using biogeneric copy tool.

On 60 randomly assigned samples, a digital impression was taken including all parts of the model as well as the post space preparation inside the canine by an IOS (Primescan, Dentsply, Bensheim, Germany). This IOS was proven to be particularly suitable for digital post scan because of its high depth of focus, brightness and light recording capability [8,22,31,32]. When superimposing the dataset for model printing, the digital construction for CPC and CAD/CAM PC was conducted using a biogeneric copy tool considering the space necessary for the telescopic crown (TC) (Figure 2).

CPCs were fabricated by milling (MCXL, Dentsply, Bensheim, Germany), using the dataset of digital construction of PCs, in a residual-free burnable resin (Telio CAD, Ivoclar, Schaan, Liechtenstein) and casting into non-precious alloy (Wirobond C, BEGO, Bremen, Germany).

**Figure 3:**
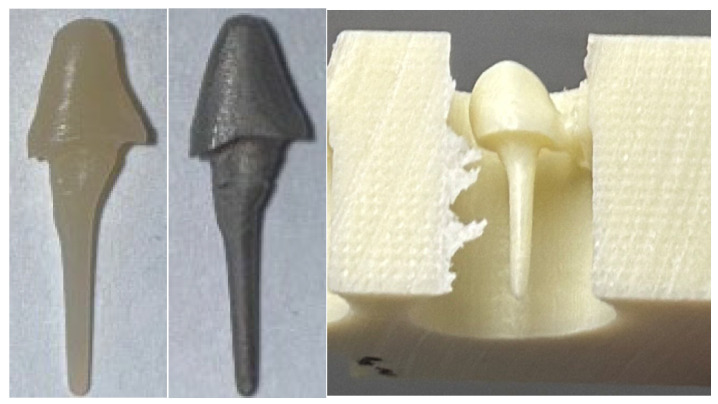
CPC: Residual-free burnable resin PC and cast PC (left). CAD/CAM PC: Milled PC made of glass fibre-reinforced composite (right).

CAD/CAM PCs were fabricated by milling, using the dataset of digital construction of PCs, in a glass fibre-reinforced composite (Trinia, Bicon, Boston, USA). Figure 3 shows the CPC and CAD/CAM PC.

CPC and CAD/CAM PC were fitted in the root canal using a resin composite (Panavia V5, Kuraray, Tokyo, Japan) according to the manufacturer’s recommendations.

**Figure 4:**
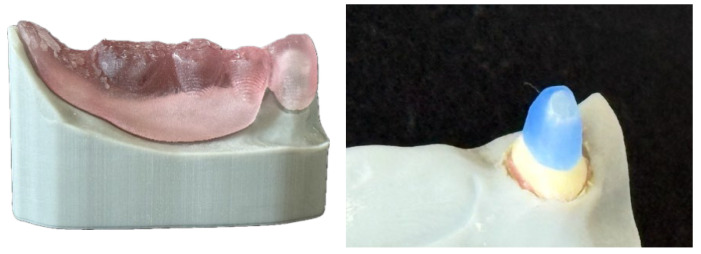
Transparent replica of TCD (left) as a template for core build-up of PFPC (right).

PFPCs (ER-Post, Komet, Lemgo, Germany) were fitted in 30 randomly assigned samples by using the same protocol as the other investigation groups. Additionally, the core was reconstructed with composite filling material (Rebilda LC, Voco, Cuxhaven, Germany). In order to keep the consistency of CPC and CAD/CAM PC in the core dimension, the dataset of TCD was used for 3D-printing a transparent replica of the TCD which was used as a template for core build-up (Figure 4).

**Figure 5:**
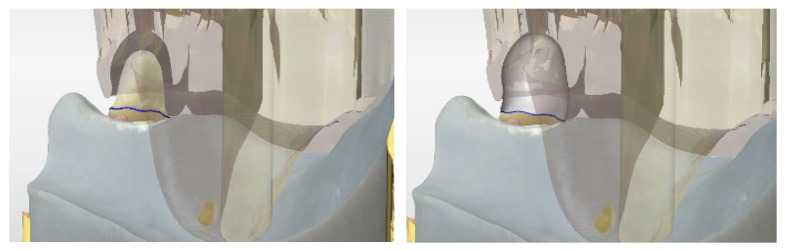
Digital impression (left) and virtual design of TC (right).

Subsequently, all 90 canines with the fitted PC were prepared for TC using diamond burs with water cooling. The preparation margin was set 2.0 mm below the post–tooth junction in order to achieve a ferule design of TC. Digital impressions of the samples were taken and TC was designed virtually in IOS software (CEREC SW 15.2) (Figure 5).

According to the fabrication of CPC, all 90 TCs were milled in residue-free burnable resin, cast in non-precious alloy and were fitted on the corresponding canine with resin composite following the same protocol as with the PC. After fitting of TC, the post material was not visually recognizable to the operator. Thus, blinding was done in order to minimize operator bias.

**Figure 6:**
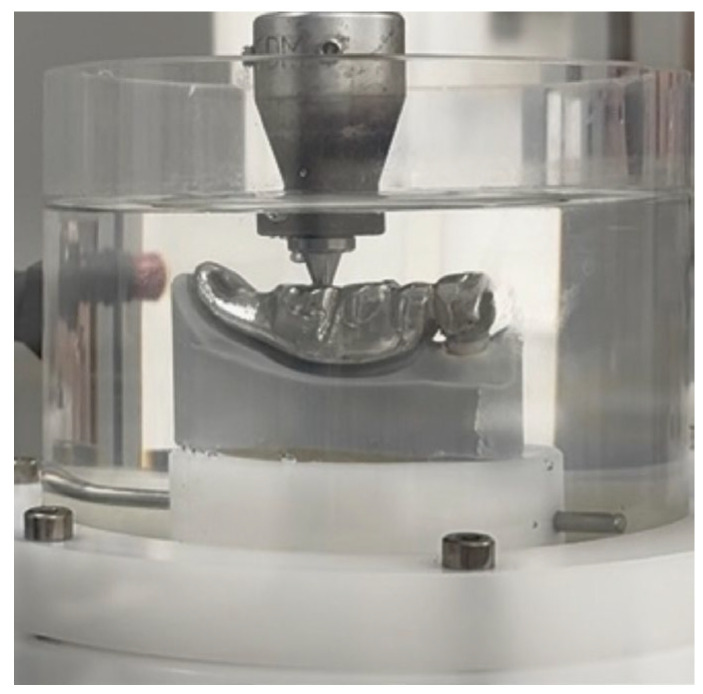
Incongruent TCD sample for chewing simulation: force on 46 results in non-axial force on the abutment tooth and the fitted PC.

In order to construct a set-up of an incongruous TCD for chewing simulation, the TCD was milled in non-precious alloy with an offset of 0.5 mm in the area of the denture saddle. Figure 6 shows the prepared sample for chewing simulation in which a force was applied on denture tooth position 46, resulting in a non-axial force on the abutment tooth and the fitted PC.

The chewing simulation with thermocycling in a water bath (SD Mechatronik Chewing Simulator CS-4.8; SD Mechatronik GmbH, Feldkirchen, Germany) was conducted for 1.2 million load cycles (Figure 6), which equals a clinical wear of 5 years [33]. The samples were randomly assigned to chewing simulation in which eight samples were tested simultaneously. After one year of simulated wear (240,000 cycles, T1–T5), the machine was stopped and the canine was analyzed for decementation and fracture using a digital light microscope. Therefore, the canine was pulled out of the impression material and was clearly repositionable after the inspection. In case of a decementation or fracture, the simulated survival time (T1–T5) was recorded in IBM SPSS Statistics for Windows, version 26 (IBM Corp., Armonk, NY, USA), and the sample was excluded from further investigation. Subsequently, after T5, the samples were assigned to the three investigation groups by another operator according to the sample number that was not known by the operator who performed the analysis for decementation and root fracture.

Decementations and root fractures during chewing simulation were evaluated by Kaplan–Meier analysis. Cases without failure were rated as “censored cases”. The pairwise comparison between the three investigation groups was performed using the Chi-square test in the form of Fisher’s exact test. *p*-values were corrected using the Bonferroni method because of alpha error accumulation. The level of significance was determined at *p* < 0.05.

## 3. Results

Two out of 30 CPCs lost retention during chewing simulation (one after T3 and one after T4). Moreover, two canines in group CPC showed a root fracture during chewing simulation (one after T3 and one after T5). Thus, the simulated 5-year survival rate in group CPC was 86.7%. Regarding PFPC, four post decementations were recorded (one after T1, two after T4 and one after T5) and two canines developed a root fracture during chewing simulation (one after T3 and one after T4). Thus, the simulated 5-year survival rate was 80.0%. Neither root fractures nor decementations were recorded during the 5-year chewing simulation of 1.2 million cycles in group CAD/CAM PC. Therefore, the survival rate in this group was 100%.

The pairwise comparisons showed a significant difference between PFPC and CAD/CAM PC (*p* = 0.02) and a barely non-significant difference between CPC and CAM/CAM PC (*p* = 0.056). Between CPC and PFPC no significant difference was found (*p* = 0.186). Table 2 illustrates the results in tabular form.

**Figure 7:**
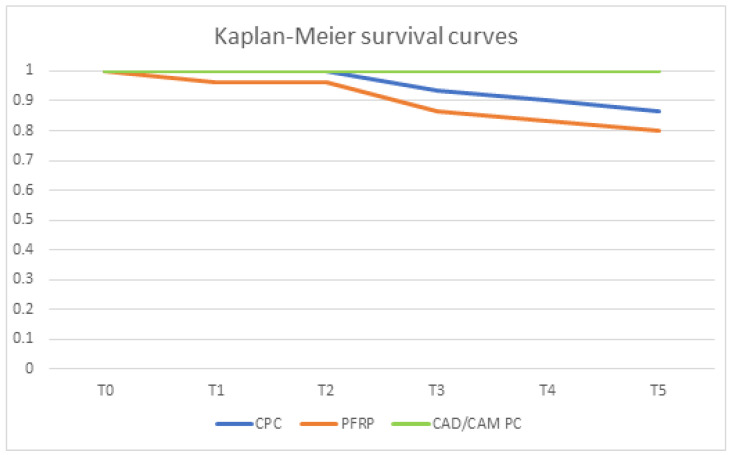
Kaplan-Meier survival curves of all investigation groups.

The Kaplan–Meier survival curves for CPC, PFPC and CAD/CAM PC are shown in Figure 7.

Summarizing the results, both null hypotheses have to be refuted because CAD/CAM PC showed significantly better performance regarding root fracture and decementation.

## 4. Discussion

Although the alloy for CPC fabrication has an elastic modulus that is far higher than that of root dentin [34,35], CPC is still the “gold standard” for treatment of teeth with a high grade of coronal destruction because of the high accuracy of fit in the root canal [4,13]. PFPCs show dentin-like mechanical properties but are less susceptible to fracture because of the interface between the post and the filling plus the poor accuracy of fit in most cases [9,13,15,35,36]. Furthermore, the use of PFPC is increasing in dental practices [37,38]. In order to eliminate the disadvantages of CPC and PFPC, CAD/CAM PCs—subtractively manufactured from a fibre-reinforced composite block with dentin-like mechanical properties—have been introduced recently [8,13,22,24]. Therefore, a comparison of these three types of PCs is of high clinical interest. One possible limitation of the present study might be that only a single brand of fibre-reinforced composite for CAD/CAM PC was used. This limits the generalizability of the results. Future investigations would benefit from a broader material scope, including PEEK or Zirconia, to provide a more comprehensive base. Moreover, the addition of a negative control group in the form of endodontically treated teeth without any post would be an opportunity to create a baseline for how these specific extra-axial forces affect the tooth structure. This might be another limitation of the present study.

For CAD/CAM PC, a digital impression of the prepared root canal is essential because subtractive manufacturing, as well as the use of fibre-reinforced composite, is limited to a digital workflow [13,39]. Recent technical developments enabled digital post scans with IOS [40] or on the basis of a scanned stone model [13]. However, since all systems on the dental market are optical systems in which light has to be reflected from every part of the root canal [41], the quality of the impression is affected by the software as well as the hardware of the scanning system [31]. An older in vitro study reported that IOS is not capable of scanning deep post space preparations [42], whereas a recent in vivo study described even better results for digital post impressions compared to analogue impressions [22]. This finding is in line with Rezaee et al. who reported superior precision for posts in a fully digital workflow [40]. That shows the significant technical development in IOS and emphasizes the need for clinicians to keep up to date. A comparison between three different IOSs showed that Primescan has the best preconditions for accurate post scanning [31]. Therefore, we chose Primescan for usage in the present study.

Furthermore, many patients with few remaining teeth are treated with TCD [1,2], but PCs under TCDs show the worst survival probability compared to all other prosthetic restorations [4,5]. Therefore, we chose a set-up imitating the common clinical situation of an incongruent TCD in order to test the three different PC types and to evaluate if CAD/CAM PCs have an advantage with TCDs as well [8].

The retention of the PC in the root canal and the material for PC fabrication is highly affected by changing temperatures and the oral environment in general [43,44]. Thus, in order to further develop new treatment options and in terms of material fatigue, preclinical load simulation in comparable set-ups to the clinical situation is essential [45]. That is why many studies described chewing simulators as suitable for evaluating the mechanical behaviour of new materials. These procedures are comparable to the method in the present study [45,46]. Furthermore, Rosentritt et al. reported that a simulation of the periodontal ligament is essential for chewing simulation because an omission leads to three times lower susceptibility to fracture, decreasing the comparability to clinical set-ups [47]. Therefore, in many studies, impression material was used for artificial periodontal ligament simulation [33,47,48] as described in the Materials and Methods section of the present study as well.

Regarding the evaluation of decementation and root fracture during load in the chewing simulation with thermocycling, both CPC and PFPC showed root fracture and decementation. For CAD/CAM PC, neither decementation nor root fracture was detected. This result is in line with the findings of a previous study investigating the performance of CAD/CAM PC for severely destroyed anterior teeth that are faced with non-axial forces too [8]. In an in vitro investigation by Hayashi et al. the fracture resistance of endodontically treated teeth with PFPC or prefabricated metallic posts was evaluated in a set-up with non-axial forces. They reported significantly higher fatigue resistance for PFPC-treated teeth and concluded that the matching elastic modulus between dentin and post material is likely to decrease the fracture rate [49]. This is partially in line with the findings of the present study but PFPC performed worse than CAD/CAM PC. One reason for the difference in the findings could be the differing set-up because in the present study, lever forces were loaded on the canine. Thus, not only the elastic modulus might be of relevance for the performance of PC but also the accuracy of fit. Bad accuracy of fit is associated with uneven transmission of force leading to fracture and decementation when non-axial forces occur [13,15,16]. In a retrospective in vivo study by Ferrari et al. the survival of CPC and PFPC was investigated over a follow-up period of up to four years. The authors described that in the PFPC group, no fracture was recorded, whereas in 9% of cases with CPC, fractures occurred [50]. Altitinchi et al. investigated CAD/CAM PC made of Trinia in a chewing simulator with thermocycling and recorded fractures in 100% of the cases. This is contrary to the findings of the present study. However, they did not consider the fibre orientation in relation to the load direction as in the present study [45]. The influence of the fibre mats in regard to the force was described in an in vitro study by Suzaki et al. and is of particular importance for PC fabrication [51]. This might be the reason for the opposing findings of Altinchi et al. and the present study regarding CAD/CAM PC made of Trinia. The majority of studies focusing on the survival of PC found decementation as the most frequent reason for failure [9,52]. This is in line with the findings of the present investigation. Decementation of PC is often characterized as a repairable failure because recementation is often possible [20]. However, the risk for extraction is significantly influenced by the decementation rate [53]. Therefore, we recorded decementation as failure but distinguished between root fracture and decementation in the results.

## 5. Conclusions

The results of the present study show that CAD/CAM PC can have an advantage over CPC and PFPC in the clinically common set-up of an incongruent TCD. Therefore, within the limitations of this in vitro study, CAD/CAM PC made of fibre-reinforced composite can be recommended for PC in abutment teeth of TCD.

## Figures and Tables

**Table 1:** Materials for CPC, PFPC and CAD/CAM PC with elastic modulus in relation to dentin.

	Materials	Product Name (LOT Number)	Brand	Elastic Modulus
CPC PFPC CAD/CAM PC	Non-precious alloy Glass fibres/epoxy resin Glass fibre mats/composite	Wirobond C (5127) ER post (235900) Trina (214711123)	BEGO Komet Bicon	180 Gpa 30 GPa 18.8 Gpa (fibres perpendicular to axis of load)

Elastic modulus of dentin: 18.6 GPa [24].

**Table 2:** Results for survival and pairwise comparisons after chewing simulation.

	Survival Rate	CPC	*p*-Values PFPC	CAD/CAM PC
CPC PFPC CAD/CAM PC	86.7% 80.0% 100%	/ 0.186 0.056	0.186 / 0.02	0.056 0.02 /

## Data Availability

The data presented in this study are available on request from the corresponding author due to privacy and ethical restrictions.
